# First Things First: How to Elicit the Initial Program Theory for a Realist Evaluation of Complex Integrated Care Programs

**DOI:** 10.1111/1468-0009.12543

**Published:** 2021-11-23

**Authors:** ROWAN G. M. SMEETS, DORIJN F. L. HERTROIJS, FERDINAND C. MUKUMBANG, MARIËLLE E. A. L. KROESE, DIRK RUWAARD, ARIANNE M. J. ELISSEN

**Affiliations:** ^1^ Faculty of Health, Medicine and Life Sciences Maastricht University; ^2^ Department of Global Health, University of Washington

**Keywords:** delivery of integrated health care, realist evaluation, program evaluation, primary health care

## Abstract

**Policy Points**

- Realist evaluation (RE) is an emerging and promising research approach for evaluating integrated care, addressing what works, how, for whom, and in what circumstances.
- The rich philosophical foundation of RE, critical realism, can help to systematically unravel an integrated care program's initial theory prior to implementation, as a first step within RE.
- RE can be considered a robust methodological asset in integrated care research by facilitating a deeper level of insight into program functioning than traditional forms of evaluation do and by shaping a realist‐informed monitoring and evaluation process.

**Context:**

The complexity of integrated care and the need for transferable evaluation insights ask for a suitable evaluation paradigm. Realist evaluation (RE), underpinned by the philosophy of critical realism, is a theory‐driven approach that addresses what works, how, for whom, and in what circumstances. The current study illustrates the process needed for RE's first step: eliciting the initial program theory (IPT). The TARGET program, a Dutch primary care initiative to facilitate more integrated care for chronically ill patients, i.e., care that is efficient, tailored, and holistic,  was taken as a real‐world case.

**Methods:**

An RE approach informed the phased IPT elicitation: (1) identifying an abstract theory framework; (2) formulating the preliminary IPT, building on the abstract theory and informed by previous scientific studies that underpin TARGET; and (3) refining the preliminary IPT, informed by RE expert interviews (*n* = 7). An RE heuristic tool, specifying the interplay between intervention‐context‐actors‐mechanisms‐outcomes (ICAMO) and retroductive reasoning, was applied to synthesize the underlying theory of individual TARGET components into TARGET's IPT.

**Findings:**

Separate but related IPTs were identified for the two main types of actors involved in TARGET: primary care professionals (PCPs) and patients. For both actors, two sorts of mechanisms are assumed to be activated by TARGET, which—via instrumental outcomes—contribute to long‐term quadruple aim targets. The first is confidence to enhance PCPs’ person‐centered conversational skills and to increase patients’ active engagement in TARGET. The second is mutual trust, between PCPs and patients and between PCPs and their network partners. A supportive context is assumed crucial for activating these mechanisms—for example, sufficient resources to invest in integrated care.

**Conclusions:**

Although the IPT elicitation process is time intensive and requires a mind shift, it facilitates a deeper insight into program functioning than accommodated by the prevailing experimental designs in integrated care. Furthermore, the design of a realist‐informed evaluation process can be informed by the IPT.

For more than a decade, scientific studies investigating the epidemiology of chronic disease have drawn notable conclusions: we face a worldwide “chronic disease epidemic” and “health care crisis.”^1^, ^2^, ^3^ To illustrate this, a recent Global Burden of Disease study concluded that noncommunicable diseases, such as diabetes and respiratory illnesses, were responsible for 73% of deaths around the globe in 2017.^2^, ^4^ This epidemic puts tremendous pressure on the sustainability of health care systems. Hence, policymakers and health care providers need to seek strategies that organize and deliver care efficiently with high responsiveness to the needs of people living with chronic diseases.^1^, ^3^, ^5^

A widely used strategy to accommodate the high burden of chronic diseases entails adopting an integrated care approach.^6^, ^7^, ^8^, ^9^, ^10^ While various definitions of integrated care exist, their common thread is that integration—that is, combining parts to form a whole—is used as a vehicle to enhance care.^6^, ^7^, ^8^, ^9^, ^10^ From a health systems’ perspective, integrated care is generally characterized by services that are managed along a continuum, coordinated across levels of care, and adapted to patients’ personal needs.^6^, ^9^, ^10^ When appropriately implemented, integrated care can contribute to the quadruple aim: improving patient experiences, the work life of health care professionals, and population health, while reducing per capita costs.^7^, ^11^ Presumably, the growing population with multimorbidity will benefit most from integrated care, as they generally require care from multiple disciplines.^12^

Despite receiving widespread support, the evidence base underpinning the effectiveness of integrated care programs remains limited and inconclusive.^13^, ^14^ One reason for the limited evidence base relates to inadequate evaluation design choices for these programs. A “pervasive belief in a hierarchy of evidence”^15^ often drives researchers to prefer traditional experimental evaluation research designs.^13^, ^14^, ^15^, ^16^ However, there is increasing awareness of the shortcomings of experimental designs, in particular for complex programs.^15^, ^16^, ^17^, ^18^, ^19^ Integrated care programs are considered complex because they require inputs from and interactions between multiple stakeholders, have several interacting program components, and are contingent on the interconnectedness with the health systems and policy environment to work successfully.^6^, ^7^, ^8^, ^13^, ^20^ Experimental designs assume a simple linear model of causality, thus focused on what works in relation to the achieved outcomes. Although this approach can be valuable for “simple” interventions, they are of limited value for interventions of a complex nature such as integrated care programs.^15^, ^21^ An appropriate evaluation for complex interventions such as integrated care should not only focus on what works but also provide answers to why, for whom, and under what conditions. Answering these questions could contribute to the current evidence base on integrated care by opening the black box for implementers about how an integrated care program achieves its outcomes and which health systems and policy conditions are conducive.^15^, ^16^, ^19^

Critical realism offers a suitable research paradigm for uncovering rich and transferable insights into the effects of integrated care programs, including their causal mechanisms and contextual influences.^22^, ^23^ Realist evaluation (RE), a theory‐driven approach to program evaluation underpinned by the critical realist philosophy of science, supports the collection of context‐linked insights to enhance program implementation.^24^, ^25^, ^26^, ^27^ The first phase of RE is to elicit the initial program theory (IPT), an underlying assumption of what works in the program, how, for whom, and in what circumstances.^24^, ^26^ Eliciting the IPT is a crucial but challenging step in RE and, although support for the use of RE for integrated care evaluation is growing, there is little practical guidance on how to elicit a robust IPT.^26^, ^28^ Therefore, we aim to provide insights into the required phased process for eliciting the IPT for an integrated care program in RE. We used the Dutch integrated care program TARGET^29^ (Targeting Advanced Resources in General practice to create Efficient, Tailored and holistic care for chronically ill patients) as a real‐world case to illustrate this process.^30^

## Real‐World Case: The TARGET Program

**Figure 1:**
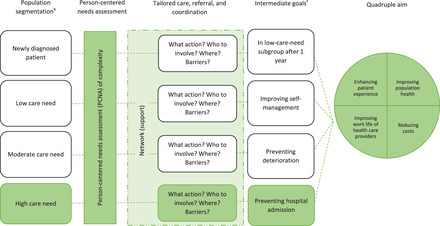
Framework of the TARGET Integrated Care Program^a^. [Colour figure can be viewed at wileyonlinelibrary.com] ^a^ The TARGET integrated care program will initially be implemented for the high‐care‐need subgroup only, highlighted in green. ^b^ The population segmentation will include all chronically ill patients suffering from at least 1 of 13 common chronic conditions: anxiety disorder, asthma, atrial fibrillation, overworking/burnout, cancer, chronic neck and back complaints, cardiovascular diseases, chronic obstructive pulmonary disease, dementia/Alzheimer's disease, diabetes mellitus, migraine, mood disorder, and peripheral arthrosis. ^c^ For each subgroup, various intermediate goals can be determined.

TARGET (see Figure 1) was developed in close cooperation with Dutch primary care. The program was theoretically inspired and informed by various scientific studies.^30^, ^31^, ^32^, ^33^, ^34^ TARGET is implemented and evaluated in Dutch general practice from 2020 until 2023. Due to the complex nature of the program, the evaluation of TARGET follows the principles of RE.^17^, ^18^, ^35^

### The TARGET Program Geographical Setting

The TARGET program is implemented in Drenthe, which is a northern, predominantly rural province of the Netherlands. Similar to other Dutch rural regions, Drenthe is confronted with a rapidly aging population that leads to high demands for care.^36^ At the same time, young general practitioners (GPs) prefer to settle in urban, more densely populated regions of the country.^37^ Hence, this province expects an alarming primary care workforce shortage in the short term.^38^ This shows the urgency for this region to invest in an efficient, integrated system of care in order to preserve the quality and accessibility of primary care. In response to this, the primary care group Huisartsenzorg Drenthe (HZD) commissioned authors RS, DH, MK, DR and AE of the current study to develop an integrated care program.^39^ Primary care groups unite and consist of various care professionals, primarily GPs.^40^, ^41^, ^42^ They were introduced in the Netherlands during the second half of 2000 and the majority of Dutch general practices are currently connected to a care group.^41^ In short, care groups support general practices in delivering disease‐specific, standardized chronic care programs for a number of conditions each under a bundled payment system (BPS). The latter means that “the price for the bundle of services (for instance, for diabetes) is freely negotiated by insurers and care groups.”^43^, ^44^ Hence, care groups represent affiliated professionals and promote their interests by functioning as the contracting party of bundled payment agreements every year. The aspects of chronic care as described in the standardized programs are delivered either by the care group itself or by other care providers (for instance, physical therapists of dietitians) who are subcontracted by the care group.^43^, ^44^ Further details on the role of care groups in the Dutch health system can be read elsewhere.^43^, ^44^, ^45^

The content of chronic care programs is determined by health care standards, which define minimum requirements for high‐quality care and specify criteria for improvements.^45^ Currently, the primary care group HZD facilitates the delivery of chronic care programs under this BPS for patients suffering from type 2 diabetes, chronic obstructive pulmonary disease, cardiovascular risks, and heart failure, separately. In addition, there is a chronic care program exclusively targeting frail elderly.^46^

### Description of the TARGET Program

TARGET aims to create integrated care, i.e., care that is efficient, tailored, and holistic, for chronically ill patients suffering from at least 1 of 13 common chronic conditions in the HZD region (see Figure 1). The program includes three main program components: (1) population segmentation; (2) person‐centered needs assessment (PCNA); and (3) network support. It is assumed that by integrating and streamlining these program components, the TARGET program will contribute to achieving the quadruple aim in the long term.^11^

The population segmentation tool, TARGET's first program component, serves to allocate all eligible chronically ill patients to one of four mutually exclusive subgroups (see Figure 1). Patients who are chronically ill for less than 12 months are allocated to the newly diagnosed patient subgroup. For patients who are chronically ill for at least 12 months, their subgroup is—in agreement with one of our previous studies^30^—determined based on the number of weighted primary care consultations in the past year: 0‐10, 11‐20, and more than 20 weighted consultations per patient per year to be assigned to the low‐, moderate‐, and high‐care‐need subgroup, respectively.^47^, ^48^ The segmentation as conducted by the tool is visualized for the primary care practices in a digital environment.

The second program component of TARGET is a yearly PCNA for patients allocated to the high‐care‐need subgroup. TARGET initially focuses on these patients to keep the implementation of the program feasible by targeting patients who presumably benefit most from TARGET due to their complex biopsychosocial needs.^33^, ^34^ The aim of the PCNA is to enhance primary care professionals’ (PCPs) insight into these needs. This insight is needed for PCPs to engage in shared decision making with patients during the PCNA about the required tailored care, referral, and coordination. In this shared decision‐making process, the following care‐related aspects need to be addressed: nature of care/support to be provided, who to involve in this care/support, where to provide this care/support, and assessment of potential barriers to obtaining this care/support. To conduct the PCNA, an expanded consultation of 30 to 45 minutes between a PCP and a patient will be scheduled. The PCPs will be offered training to enhance their person‐centered conversational skills. In addition, they can choose between two conversation tools. The first tool is the My Positive Health conversation tool, which is based on the concept of “positive health” as introduced by Huber and colleagues.^49^, ^50^, ^51^ The second tool is the Patient Centered Assessment Method (PCAM) questionnaire and a visualization derived from the questionnaire. The latter also serves to record and evaluate the biopsychosocial complexities and possible actions.^52^

The third component of TARGET relates to the provision of support to enhance the network of PCPs: enhancing the insight into, as well as communication and cooperation with, the network. After all, if the PCNA revealed that the patient's needs should be primarily dealt with elsewhere, referral will be facilitated only if a strong network has been composed. Relevant disciplines to be included in this network are, among others, mental health care, community nursing, and social care. The combination of these three program components is assumed to help PCPs to realize the determined tailored care, referral, and coordination, as an intermediate outcome for achieving quadruple aim targets.

## Methodological Approach

RE, introduced by Pawson and Tilley, is a theory‐driven evaluation approach philosophically underpinned by critical realism.^19^, ^24^, ^27^, ^35^ One of the tenets of critical realism relates to the understanding that, in society and social activity, both social structure (i.e., the organized set of social institutions and patterns of institutionalized relationships) and agency (i.e., thoughts and actions taken by people) play a key role.^53^, ^54^ Structures as well as agents possess generative or causal powers, which are important to consider in understanding and explaining social behavior and change. More specifically, as described by Elder‐Vass, “critical realist social theory recognizes that both human individuals and social structures (and indeed entities of other kinds) have causal powers that are distinct from each other, and that both (or all) interact to determine social events—even though human individuals are the parts of the social structures concerned.”^55^

Mukumbang and van Wyk^54^ argued that these powers only come about and lead to events when certain latent mechanisms are activated under the right conditions. For example, only if a team meeting generates a feeling of belongingness (the mechanism driving change) among team members, then better communication and cooperation are potentially achieved.^54^ Because of the importance of generative mechanisms in explaining the occurrence of certain events, critical realist efforts are highly focused on their elicitation. However, traditional and direct empirical methods are often unsuited for understanding these latent mechanisms. Rather, “a combination of empirical investigations and theory construction” is needed.^56^ In addition to this, Mukumbang and van Wyk^54^ describe that, from a critical realist philosophy, the activation and effects brought about by mechanisms are contingent on contextual conditions.^54^ See the Appendix for a more elaborate discussion of the methodological differences between RE and prevailing approaches.

Corresponding to this central understanding of context‐dependent mechanisms, RE traditionally uses the context‐mechanism‐outcome (CMO) configuration heuristic tool to support theory development.^19^, ^57^, ^58^, ^59^ This tool is used to illustrate how under certain conditions (C), naturally occurring mechanisms (M) or those provided by an intervention in the target population are activated to produce certain behaviors or outcomes (O). The RE literature shows small differences in the definitions of *context*, *mechanism*, and *outcome*. In the current study, we used the definitions as presented in a recent study by Mukumbang and colleagues^58^ (Table 1), which correspond to the work of Pawson and Tilley.^59^ In addition, the traditional CMO heuristic tool of Pawson and Tilley is expanded in the present study by adding “intervention” (I) and “actors” (A), as proposed by Mukumbang and colleagues and Marchal and colleagues.^60^, ^61^ This results in the ICAMO heuristic tool that will be used throughout this paper. After all, it can be argued that the degree to which outcomes (O) are achieved—by triggering mechanisms (M) under the right conditions (C)—is dependent on the degree to which the intervention (I) is successfully delivered and adopted by the various actors (A) who are involved in the implementation. The elements of the ICAMO tool, which are defined in Table 1, can be illustrated by the following simple example:

**Table 1:** Definitions of the Elements Included in the ICAMO Heuristic Tool

Element of ICAMO Heuristic Tool		Definition
Intervention		A combination of program elements or strategies, in particular, those designed to produce behavior changes or improve health status among individuals or a group
Context		The salient conditions that are likely to enable or constrain the activation of program mechanisms
Actors		The individuals, groups, and institutions that play a role in the implementation and outcomes of an intervention
Mechanisms		Any underlying determinants of social behavior generated in certain contexts
Outcomes	Immediate	The immediate effect of program activities
	Intermediate	Behavioral changes that follow the immediate knowledge and awareness changes
	Long term	Changes in the medium and long term, such as a patient's health status, and impact on community and health system

Data retrieved from Mukumbang et al.^58^

Regular team meetings (I) organized by a general practitioner (A) at an inspiring location (C) could give team members (A) a feeling of belongingness (M), potentially leading to better communication and cooperation within the team (O).

## Methods

**Figure 2:**
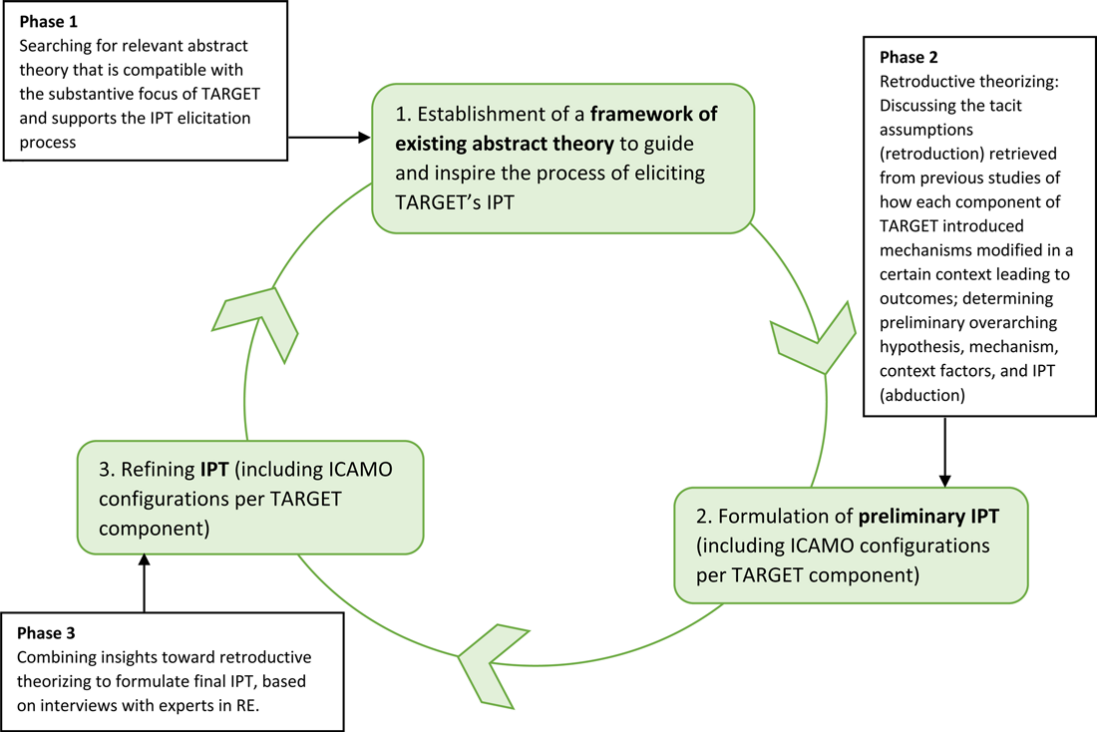
The Phased Strategy to Eliciting the IPT for the TARGET Integrated Care Program [Colour figure can be viewed at wileyonlinelibrary.com]

We used the ICAMO heuristic tool and retroductive theorizing to elicit the IPT of TARGET. An IPT process consisting of three phases was used, as depicted in Figure 2. This process was reflective in nature, implying that the different phases informed each other forward as well as backward.

### Phase 1: Framework of Existing Abstract Theory

We chose the Comprehensive Theory of Integration proposed by Singer and colleagues to guide the IPT elicitation process.^7^ The reasons for this choice were twofold. First, this theory is compatible with the substantive focus of the TARGET program—that is, the investment in integration of care services with the aim of achieving patient‐centered integrated care. Singer defines integration as “the making of a unified whole from distinct and interdependent organizational components.”^7^ In the field of integrated care, Singer's theory is considered a seminal contribution: it is built on a synthesis of previous theoretical and conceptual efforts in integrated care.

Second, the theory of Singer identifies some elements—–constructed as a logic chain—that also have a place in the traditional RE heuristic tool and as such are critical to RE theory formulation: contextual factors, interventions (i.e., integration modalities), and intermediate and final outcomes. According to Singer and colleagues, contextual factors serve as “precursors to organizational (related to structures and systems) and social (related to norms and behavior) types of integration.”^7^ By investing in different types of integration, it is assumed that both intermediate outcomes, such as integrated patient care, and final outcomes, such as efficiency, are potentially realized.

It should be noted, however, that Singer's theory also has limitations when assessed using a critical realist lens. This mainly pertains to the generative understanding of causality in RE that is not entirely represented in this theory. It largely overlooks the role of latent, generative mechanisms, such as mutual trust and provider confidence, in explaining the outcomes of integration efforts, and it tends to mainly focus on tangible intervention modalities. Therefore, other sources were needed to unravel the generative mechanisms that impact the hypothesized functioning of TARGET (see phases 2 and 3).

### Phase 2: Preliminary IPT

In phase 2, we consulted different scientific studies in the field of integrated care, including our own previous studies.^30^, ^31^, ^32^, ^33^, ^34^ These studies, displaying tacit theory, had collectively inspired and informed the composition of TARGET. From these studies, we retrieved insights into potential intervention users (key actors), possible key contextual conditions, and desired outcomes to supplement our developing IPT from Singer's theory.

A recent publication on effective care for high‐need patients outlined the feasibility and clinical relevance of a simple stratification of high‐need patients according to the type and intensity of expected care needs.^31^ Routinely registered medical data were taken as a starting point of stratification. Subsequently, functional, social, and behavioral factors were also taken into account for each of the identified subgroups, as these factors are acknowledged as other key drivers of need. This comprehensive insight would subsequently provide guidance to determining tailored care models and care teams. In our first explorative cohort study, described next, we aimed to assess whether such a simple stratification tool, taking routinely registered data as a starting point, was equally feasible and clinically relevant for chronically ill people in the HZD region.^30^

The cohort study showed that it is possible to create a segmentation tool that classifies a heterogeneous chronically ill population into three subgroups with varying care needs: low, moderate, and high. Although each subgroup is responsible for exactly one‐third of the cumulative care utilization, the number of patients in each subgroup is significantly different.^30^ Although the low‐care‐utilization subgroup includes 63.4% of chronically ill patients, with each consuming approximately five consultations per years, the high‐care‐utilization subgroup includes 12.3% of chronically ill patients, each consuming approximately 30 consultations per year. Furthermore, each subgroup is characterized by a different set of patient characteristics associated with the level of care utilization each subgroup consumes. Hence, patients in the high‐care‐utilization subgroup have, in sharp contrast to the low subgroup, individually significantly more chronic conditions and are more likely to be older, to be female, and to have a combination of physical and mental conditions.^30^

The latent class analysis expanded on the previous explorative study by identifying, based on combinations of biopsychosocial patient characteristics, different relevant latent subgroups of high‐need, high‐cost (HNHC) chronically ill people.^33^ HNHC patients were defined as those who belonged to the top 10% of care utilizers and/or had multimorbidity accompanied with an above‐average care utilization. This study revealed that the HNHC chronically ill patient population can be divided in four latent classes. The two largest classes, together including almost two‐thirds of patients, represent older adults who mainly have physical and age‐related conditions (e.g., diabetes, osteoarthritis, and cancer). The two remaining classes, together including more than one‐third of patients, represent middle‐aged adults who more often have to deal with social welfare dependency and mental conditions (e.g., mood disorders). As such, this study underlined the need to take into account the biopsychosocial diversity of the HNHC population in tailoring care to the complex needs of these patients.

Our third previous study that informed TARGET was a qualitative one: five focus group discussions were organized with 42 PCPs.^34^ This study was inspired by the Bridges to Health model, which illustrated how priorities, components of care, and goals can be determined for previously identified subgroups of patients.^32^ Corresponding to the approach taken in this Bridges to Health study, we developed case descriptions of typical patients of each of the HNHC classes identified in our previous study. Based on these case descriptions, we initiated a discussion with PCPs on the experienced barriers and possible solutions with regards to person‐centered, efficient care delivery to each subgroup of HNHC patients. It was concluded that investment in the organization of primary care, as well as in the communication and cooperation (i.e., healthy collaborations) between primary care and other settings is needed for PCPs to effectively deal with the complex needs of HNHC patients. Thus, general practices need to be provided more consultation time, skilled PCPs, and information and communication technology solutions for efficient information retrieval and sharing. In addition to this, interdisciplinary communication and cooperation should be fostered, which could—among other outcomes—facilitate referral of primarily psychosocial patient needs to other settings.

The ICAMO configuration was used to construct the developing IPT. Authors RS, AE, and DH used several meetings to discuss the tacit assumptions of how each intervention component of TARGET shapes the mechanisms, when introduced in a certain context, and as such potentially leads to outcomes.^33^ This was achieved through retroduction, which refers to “the activity of theorizing (and testing) for hidden causal mechanisms responsible for manifesting the empirical, observable world”^62^ (see the Appendix for more information).^22^, ^62^, ^63^ Retroduction in RE should be combined with a process of abduction, resulting in so‐called retroductive theorizing. Abduction can be described as “inventive thinking required to imagine the existence of such mechanisms”^62^ and is needed in order to actually study the mechanisms as identified by retroduction.^22^, ^62^ Hence, we applied abductive reasoning by taking a step back and formulating a preliminary overarching hypothesis as well as mechanism and context factors. The preliminary IPT (including configurations per TARGET component) as formulated by authors RS, AE, and DH was discussed with authors MK and DR to reach a consensus.

### Phase 3: Refining the IPT

To formulate the final IPT of TARGET, the preliminary IPT (including configurations per TARGET component) that resulted from phase 2 was discussed with seven experts in RE over the course of five interviews: one focus group interview with three experts, and four individual interviews. A priori, we considered five interviews as sufficient to revise the preliminary IPT in a well‐informed way. The experts were selected purposively to ensure that each expert had considerable and relevant expertise in RE, preferably related to the field of integrated care or a closely related field of study. Four respondents had two to five years of experience in RE; the other three respondents had seven to ten years of experience in RE. The interviews were conducted in pairs: RS guided the interviews, and AE or DH provided support by asking follow‐up questions and taking notes during the interviews. Each interview lasted approximately one hour and was audio recorded. The secured video‐conferencing platform Zoom was used to conduct the interviews.

Before the start of the interviews, we prepared respondents by sending them an email in which we explained the reason for the interview and provided a short description of TARGET, along with the preliminary IPT (including configurations per individual component) we formulated. In addition, information was provided on the ethical procedures of the interviews, and we asked participants to return a signed informed consent form before the interview. A structured interview guide was developed, including two topics: methodological validity and substantive judgmental rationality (evaluating the explanatory power of different theoretical explanations to select theories that most accurately represent how and why the program would work).^22^, ^23^ Hence, we asked respondents to comment on the methodological validity of the preliminary IPT from the philosophical standpoint of RE, such as whether the identified mechanisms in the preliminary configurations could indeed be considered mechanisms.^35^, ^59^, ^64^ Furthermore, respondents were asked to comment on the IPT, considering their theoretical knowledge of and/or experience with the implementation of integrated care interventions. By applying judgmental rationality one can unearth better or worse arguments on behalf of elicited theories.^22^, ^23^

After the last interview, authors RS, AE, and DH discussed whether theoretical saturation was reached and assessed whether additional interviews were needed. Theoretical saturation was determined by considering the degree to which interviews still provided reason to change the direction or consistency of the developing program theory.^27^, ^65^ Based on the criterion of theoretical saturation, we decided that no additional interviews were needed.

The ICAMO heuristic tool and the logic of retroduction guided the theory‐refining process. The preliminary IPT was refined in a stepwise manner, based on the insights obtained from the interviews. First, author RS combined all insights of the interviews into a data matrix. In this matrix, the main comments, which were categorized as related to either “methodological validity” or “substantial relevance,” were specified for each individual preliminary configuration. This helped to identify agreements as well as discrepancies among the comments from the different experts. From these comments, we derived overarching and specific recommendations and lessons to refine our IPT, and we composed an initial draft of the refined configurations, which was discussed with all authors. After formulating the final IPT and configurations per individual component, all configurations were transformed into if…, then…, because… statements.^25^, ^66^, ^67^ More specifically, we defined “IF this intervention (I) modality is introduced for these actors (A), THEN this outcome (O) would be achieved, BECAUSE these mechanisms (M) are triggered under particular conditions (C).” These testable hypotheses, which are regarded as “the most basic format for programme theories,”^25^ aid in formulating underlying program theory in a simple, coherent, and functional way.^25^, ^66^, ^67^

## Results

### Framework of Abstract Theory

**Figure 3:**
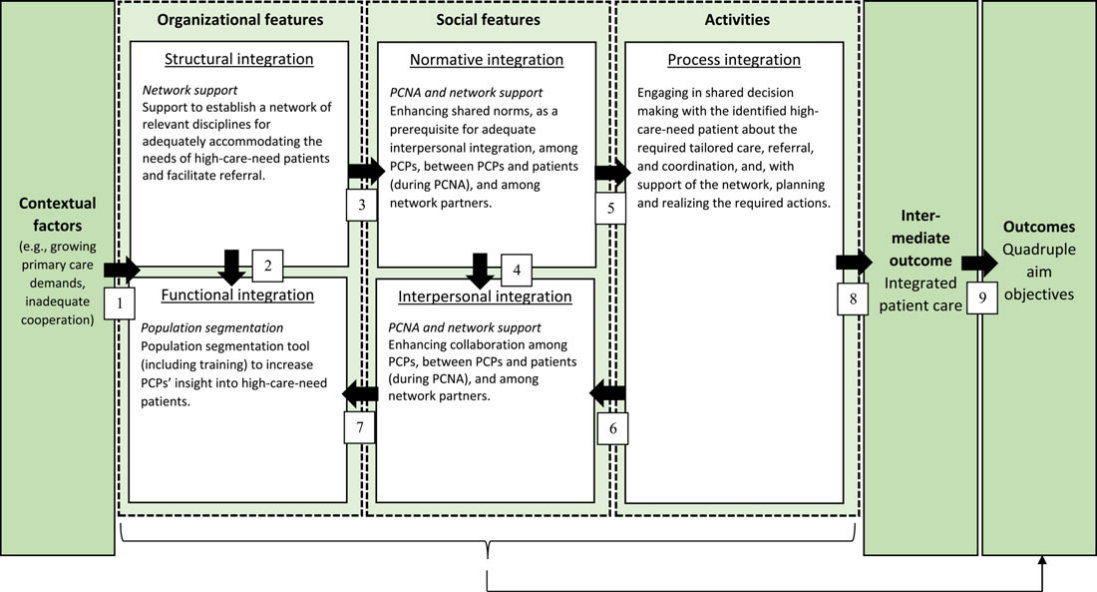
Framework of Existing Abstract Theory Underlying TARGET [Colour figure can be viewed at wileyonlinelibrary.com] Abbreviations: PCNA, person‐centered needs assessment; PCP, primary care professional. Adapted from the “conceptual model of integration types” as introduced by Singer et al.^7^

Figure 3 shows the theoretical framework that was developed to inspire the RE process, underpinned by the theory of Singer and colleagues.^7^ The arrows show the hypothetical relationships that are present according to Singer and colleagues^7^, which move from left to right, but also show directionality or feedback loops. It illustrates that the need to introduce different types of integration in the TARGET program is triggered by contextual factors, see first arrow on the left: among others, inadequate cooperation within primary care as well as between primary care and the network of relevant care and social disciplines. Introducing three program components, the TARGET program would contribute to all five types of integration: structural and functional integration (related to organizational features), normative and interpersonal integration (related to social features), and process integration (related to activities). Doing so is anticipated to contribute to integrated patient care on an intermediate level, and to reach the quadruple aim consequently (arrows 8 and 9).

By offering network support and population segmentation, TARGET aims to invest in structural and functional integration. Hence, in a more direct way, the ties between professionals within and between organizations are strengthened. The segmentation tool would do this within general practice by offering digital information about patients’ health care needs to the team of professionals. The network support is focused on creating partnerships between general practice and relevant network partners. This can help to work more functionally integrated (arrow 2). These tangible forms of integration intend to, subsequently, work toward integration on a social level (arrow 3). The PCNA and network support aim to enhance shared norms (i.e., normative integration) which strengthens collaboration (i.e., interpersonal integration) among PCPs, between PCPs and patients (during PCNA), and among network partners (arrow 4). The organizational and social forms of integration would serve as a foundation for integrating care in terms of the process (arrow 5), often referred to as clinical integration: engaging in shared decision making with the identified patients about the required “tailored care, referral, and coordination,” and, with support of the network, planning and realizing the required actions. Arrows 6 and 7 illustrate that the relationship between the different types of integration are bidirectional. Hence, stronger clinical integration would also strengthen shared norms and collaboration (related to social features). This may also enhance the network ties and valuable use of the population segmentation tool (related to organizational features).

### Hypothesized Functioning of TARGET Component 1 From the PCP perspective

**Figure 4:**
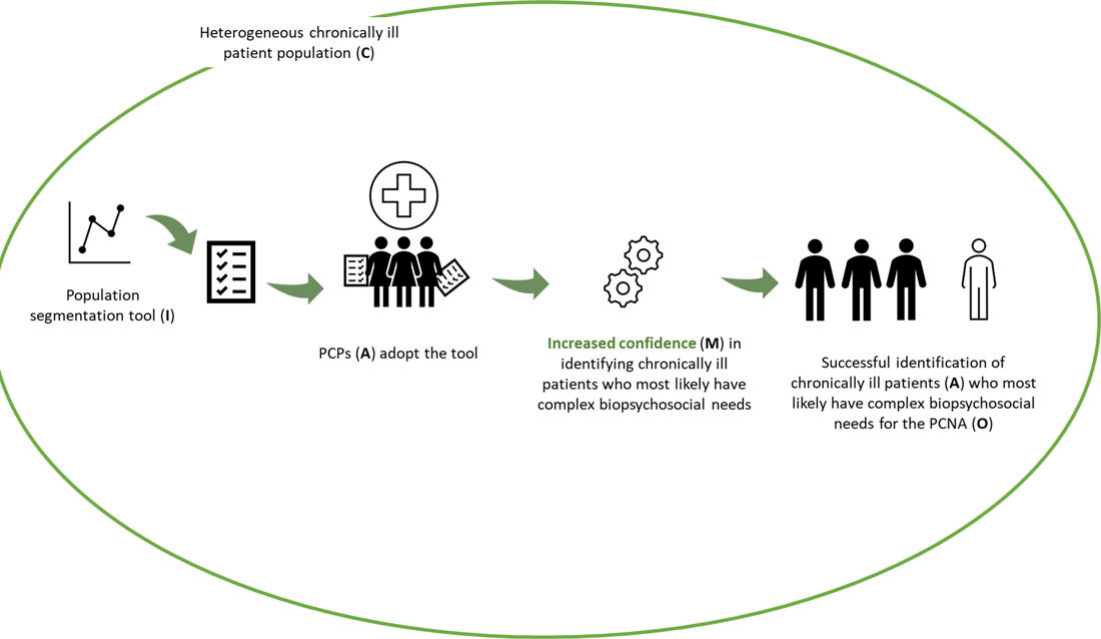
The ICAMO Configuration of the TARGET Population Segmentation Tool From the Perspective of the PCP. [Colour figure can be viewed at wileyonlinelibrary.com] Abbreviations: I, intervention; C, context; A, actor; M, mechanism; O, outcome; PCNA, person‐centered needs assessment; PCP, primary care professional.

Figure 4 illustrates how component 1 (population segmentation) would function for the PCP. A population segmentation tool (I) instills confidence (M) in PCPs (A) to successfully identify chronically ill patients who most likely have complex biopsychosocial needs for a PCNA (O) in the context of a heterogeneous chronically ill patient population (C).

Converting this theory into a testable hypothesis using the If…, then…, because… statement, we obtained the following:

**IF** a population segmentation tool is provided to PCPs,

**THEN** PCPs can successfully identify chronically ill patients who most likely have complex biopsychosocial needs for a PCNA,

**BECAUSE** PCPs gained increased confidence in identifying chronically ill patients who most likely have complex biopsychosocial needs, in the context of a heterogeneous chronically ill patient population.

### Hypothesized Functioning of TARGET Component 2 From the PCP Perspective

**Figure 5:**
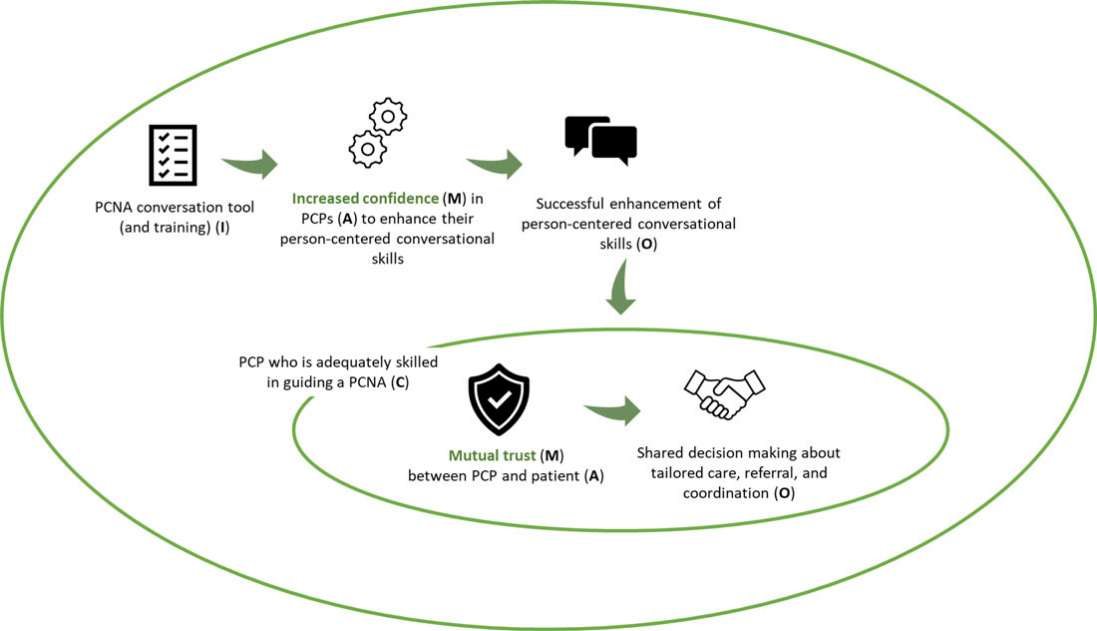
The ICAMO Configuration of the TARGET PCNA Tool, From the Perspective of the PCP. [Colour figure can be viewed at wileyonlinelibrary.com] Abbreviations: I, intervention; C, context; A, actor; M, mechanism; O, outcome; PCNA, person‐centered needs assessment; PCP, primary care professional.

Figure 5 illustrates how component 2 (PCNA) would function for the PCP. The PCNA conversation tool and training (I) instill confidence (M) in PCPs (A) to enhance their person‐centered conversational skills (O). Enhanced conversational skills (C) incite mutual trust (M) between the PCP and the patient (A), enabling shared decision making about tailored care, referral, and coordination (O).

Converting this theory into testable hypotheses using the If…, then…, because… statement, we obtained the following:

**IF** PCPs are offered a PCNA conversation tool and training,

**THEN** PCPs are likely to enhance their person‐centered conversational skills,

**BECAUSE** the tool and training instill confidence in PCPs to enhance these skills.

**THEN,** in a context where PCPs are adequately skilled in guiding a PCNA, PCPs are enabled to engage in shared decision making about the required tailored care, referral, and coordination

**BECAUSE** mutual trust is incited between the PCP and the patient.

### Hypothesized Functioning of TARGET Component 2 From the Patient Perspective

**Figure 6:**
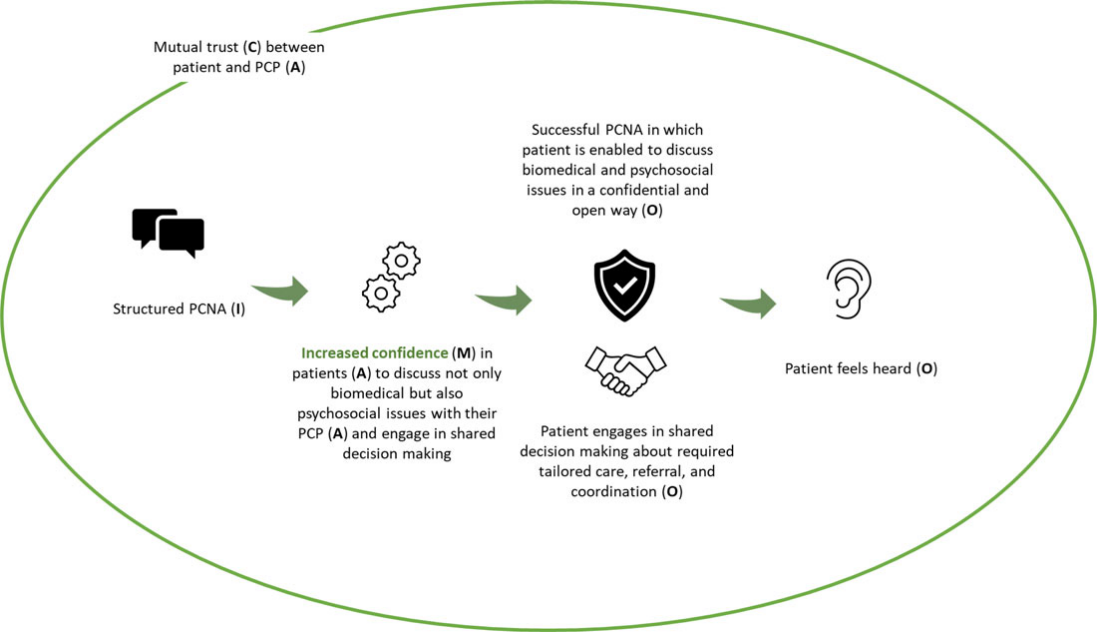
The ICAMO Configuration of the TARGET PCNA From the Perspective of the Patient. [Colour figure can be viewed at wileyonlinelibrary.com] Abbreviations: I, intervention; C, context; A, actor; M, mechanism; O, outcome; PCNA, person‐centered needs assessment; PCP, primary care professional.

Figure 6 illustrates how component 2 (PCNA) would function for the patient. Within a context of mutual trust (C), a structured PCNA (I) instills confidence (M) in high‐care‐need patients (A) to discuss not only biomedical but also psychosocial issues with their PCP (A) in a confidential and open way (O) and engage in shared decision making about the required tailored care, referral, and coordination (O). As a result, patients feel heard (O).

Converting this theory into a testable hypothesis using the If…, then…, because… statement, we obtained the following:

**IF** high‐care‐need patients are offered a structured PCNA within a context of mutual trust,

**THEN** patients are offered the opportunity to discuss their biopsychosocial issues in a confidential and open way and engage in shared decision making about the required tailored care, referral, and coordination,

**BECAUSE** confidence is instilled in patients to openly discuss their problems and engage in shared decision making. As a result, patients feel heard.

### Hypothesized Functioning of TARGET Component 3 From the PCP Perspective

**Figure 7:**
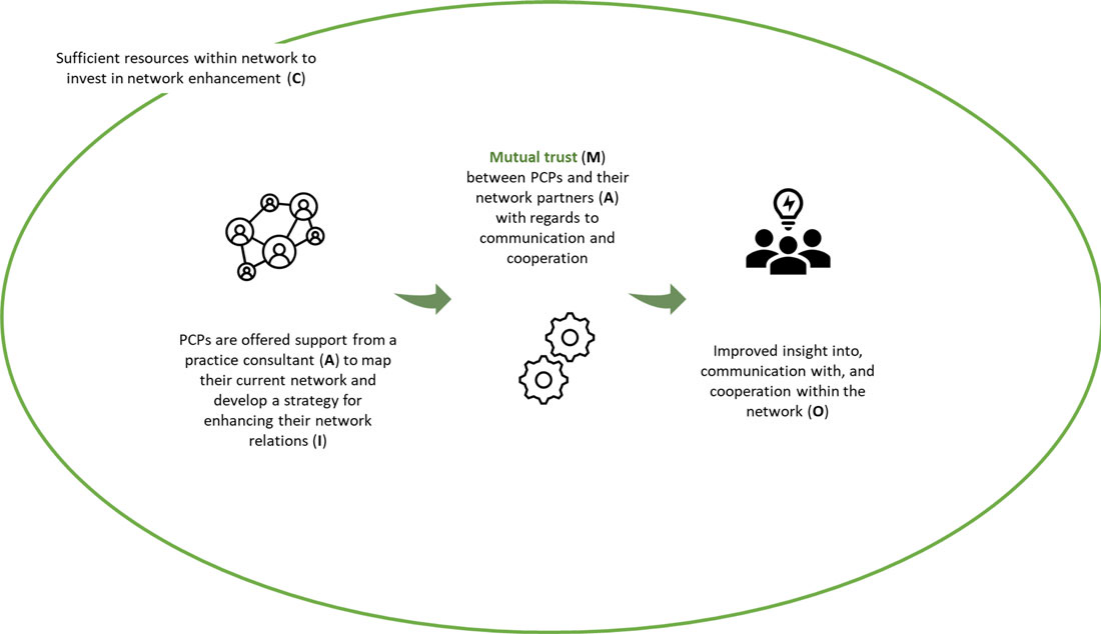
The ICAMO Configuration of the Network Support, From the Perspective of the PCP. [Colour figure can be viewed at wileyonlinelibrary.com] Abbreviations: I, intervention; C, context; A, actor; M, mechanism; O, outcome; PCP, primary care professional.

Figure 7 illustrates how the program component—network support—introduced in component 3 would function for the PCP. The support offered by a “practice consultant” (A) to map PCPs’ current network and develop a strategy for enhancing their network relations (I) would enhance mutual trust (M) between PCPs and network partners (A) with regards to communication and cooperation, in a context of sufficient resources within the network to invest in network enhancement (C). As a result, the PCPs’ insight into, communication with, and cooperation within the network is improved (O).

Converting this theory into a testable hypothesis using the If…, then…, because… statement, we obtained the following:

**IF** PCPs are offered support from a practice consultant to map their current network and jointly develop a strategy for enhancing their network relations,

**THEN** their insight into, communication with, and cooperation within the network will be improved,

**BECAUSE** mutual trust is—in a context where the network has sufficient financial resources to invest in network enhancement—incited between PCPs and their network partners with regards to communication and cooperation.

### Hypothesized Functioning of TARGET for PCPs

**Figure 8:**
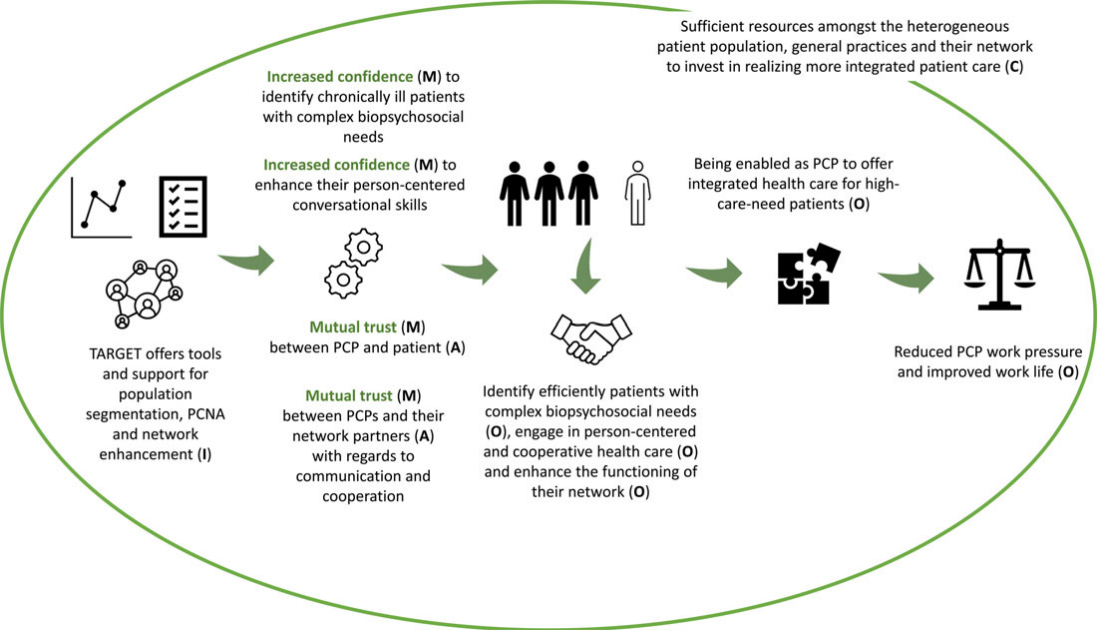
The ICAMO Configuration of TARGET's IPT from the Perspective of the PCP. [Colour figure can be viewed at wileyonlinelibrary.com] Abbreviations: I, intervention; C, context; A, actor; M, mechanism; O, outcome; PCNA, person‐centered needs assessment; PCP, primary care professional.

Figure 8 illustrates how the TARGET program would function (i.e., the overarching IPT) for the PCP. In the context of involved parties (e.g., patient population, practices, network partners) who have sufficient resources to invest in integrated patient care (C), the TARGET program offers PCPs (A), through the population segmentation and PCNA tools as well as support to enhance their network (I), opportunities and resources to identify efficiently patients with complex biopsychosocial needs (O), engage in person‐centered and cooperative health care (O), and enhance the functioning of their network (O) as these tools incite confidence (M) in the PCPs and mutual trust (M). The TARGET program, therefore, empowers PCPs to offer integrated patient care (O) to high‐care‐need patients, thereby reducing PCPs’ work pressure and improving their work life (O). In the current study, integrated patient care is defined as “care that is efficient, tailored, and holistic.”

Converting this theory into a testable hypothesis using the If…, then…, because… statement, we obtained the following:

**IF** PCPs are offered tools and support for population segmentation, PCNA, and network enhancement,

**THEN** PCPs are provided opportunities and resources to efficiently identify patients with complex biopsychosocial needs, engage in person‐centered and cooperative health care, and enhance the functioning of their network,

**BECAUSE** these tools and support incite confidence in PCPs, and mutual trust (both between PCPs and patients, and PCPs and their network partners), in a context of sufficient financial resources among all involved parties to invest in realizing more integrated patient care. As a result, PCPs are empowered to offer integrated patient care to high‐care‐need patients, thereby potentially reducing PCPs’ work pressure and improving their work life.

### Hypothesized Functioning of TARGET for Patients

**Figure 9:**
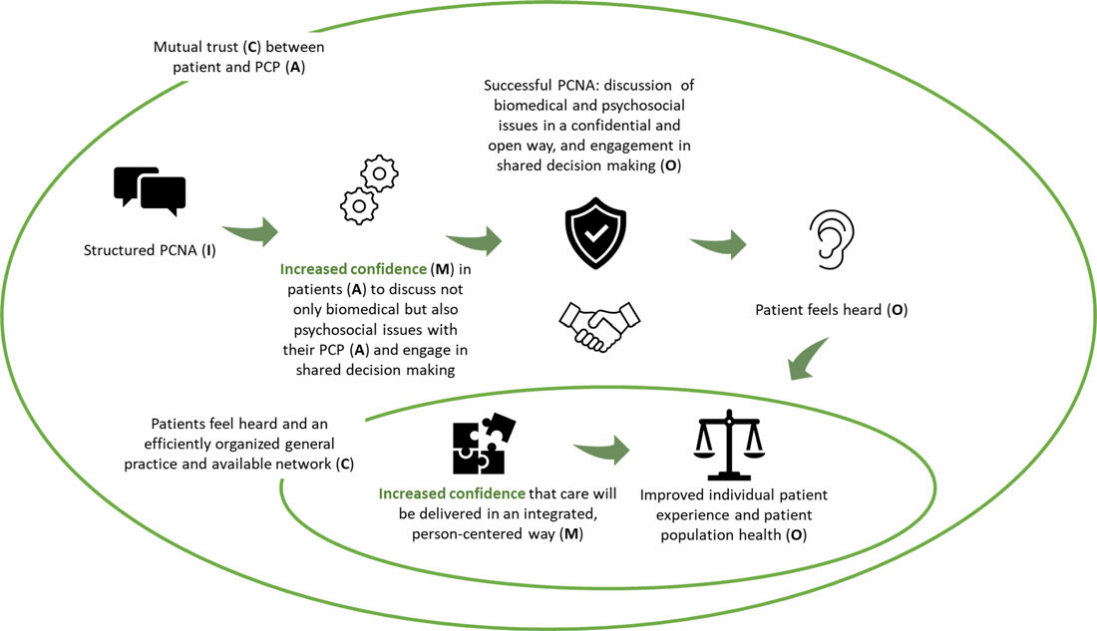
The ICAMO Configuration of TARGET's IPT From the Perspective of the Patient. [Colour figure can be viewed at wileyonlinelibrary.com] Abbreviations: I, intervention; C, context; A, actor; M, mechanism; O, outcome; PCNA, person‐centered needs assessment; PCP, primary care professional.

Figure 9 illustrates how the TARGET program would function (i.e., the overarching IPT) for the patient. In the context of mutual trust (C), the TARGET program offers patients with complex needs (A), through the PCNA (I), the feeling of being heard (O), as the PCNA incites confidence (M) in patients (A) to discuss not only their biomedical but also their psychosocial issues (O) and engage in shared decision making (O). In a context where patients feel heard and there is an efficiently organized practice and available network (C), confidence (M) is incited in patients that their required care will be delivered in an integrated, person‐centered way, thus improving individual patient experience and, in the long term, patient population health (O).

Converting this theory into testable hypotheses using the If…, then…, because… statement, we obtained the following:

**IF** high‐care‐need patients are engaged in a structured PCNA within a context of mutual trust,

**THEN** patients will feel heard,

**BECAUSE** the PCNA incites confidence in patients to discuss their biopsychosocial issues and engage in shared decision making.

**THEN** individual patient experience and, in the long term, patient population health is improved,

**BECAUSE** confidence is incited in patients that, in a context where patients feel heard and an efficiently organized general practice and network is available, their required care will be delivered in an integrated, person‐centered way.

## Discussion

In this paper, we illustrated how to derive an IPT for a complex integrated care program, as a first crucial step toward conducting an RE. The TARGET integrated care program, a Dutch primary care initiative to facilitate more efficient, tailored, and holistic care for chronically ill patients, was used as a real‐world case to illustrate this process. By adopting the ICAMO heuristic tool and inspired by retroductive reasoning, ICAMO configurations of TARGET's three individual program components were established: population segmentation tool, person‐centered needs assessment, and network support. Configurations of individual components were systematically synthesized into two separate but complementary IPTs, one for each main type of actor involved in TARGET: PCPs and patients. We identified two main mechanisms that are hypothesized to be activated by TARGET: confidence and mutual trust. These two are assumed to contribute—via instrumental outcomes—to the achievement of long‐term quadruple aim targets. It is hypothesized that these mechanisms are only activated within a supportive context—for example, sufficient resources to invest in integrated care.

The IPT identified in this study shows how different types of integration, as identified by Singer and colleagues^7^ and classified as organizational, social, and related to activities, would be enhanced by introducing the TARGET program in general practice. Although TARGET would focus on all five types of integration as deemed important by Singer, integrating in terms of social features (i.e., stimulating shared norms and cooperation) is most elaborately addressed by the program's components, as compared to the limited set of organizational integration efforts included in TARGET. Previous research suggests that organizational and social integration reinforce each other toward delivering integrated care and reaching improved outcomes, underlining the importance of investing equally in both forms of integration.^7^, ^68^, ^69^, ^70^ Hence, it is worth considering to expand the TARGET program in the future with additional organizational integration components, informed by the unfolding RE. For example, as elaborately described by Embuldeniya and colleagues,^68^ introducing an integrated funding model as a new intervention may be a valuable addition. A bundled payment model already exists in the Netherlands for various disease management programs for common chronic conditions.^43^, ^45^ However, this model is criticized for facilitating integration between only a limited number of care professions and primarily in a single setting (i.e., primary care).^71^, ^72^ As TARGET aims to facilitate collaboration and integration between a wide variety of disciplines and across health and social care settings, the current payment model would thus need revision and a broader scope to be a suitable new intervention modality.

Methodologically, this paper illustrates how the RE approach can help create insight into the often implicit theory underlying a program. In doing so, RE has the potential to reach a deeper insight into program functioning than allowed for by the prevailing evaluation approaches in the field of integrated care, which have relied on mostly controlled studies.^13^, ^14^, ^15^, ^21^, ^73^ More specifically, experimental designs with a linear model of causality have a substantially different methodological standpoint about central RE concepts, such as mechanisms and contextual drivers of change.^21^ For instance, experimental designs do not put generative mechanisms at the heart of behavior change and evaluation efforts. Rather, “experimental designs, especially RCTs [randomized controlled trials], consider human desires, motives and behavior as things that need to be controlled for.”^21^ Likewise, context is not considered a central aspect of program functioning, which would determine whether or not mechanisms fire. Hence, in experimental designs, “the influence of context will be levelled out by, for example, including study sites whose contexts are broadly comparable.”^21^

For the field of integrated care, new insights and lessons can be distilled from the presented, hypothesized IPT. First, the IPT suggests that there are certain mechanisms in TARGET that are activated under specific circumstances. These mechanisms in turn influence the different types of actors and lead to predefined outcomes. While TARGET's program components are considerably different from a substantive point of view, two types of overarching mechanisms are triggered: confidence and mutual trust. This corresponds with previous realist‐inspired work in the field of integrated care addressing *how* initiatives work.^68^, ^73^, ^74^ For instance, a realist synthesis by Tyler and colleagues identified “four consistent patterns of care that may be effective” in six unique social pediatric initiatives.^74^ Similar to our findings, these include bridging trust and practitioner confidence, among others. Another realist review, by Kirst and colleagues, also identified “trusting multidisciplinary team relationships” as one of two overarching mechanisms in 28 integrated care programs.^73^ At the same time, it should be acknowledged that evidence on crucial mechanisms in integrated care is scarce. This can be explained in part by the novelty of RE for the field of integrated care. Consequently, a generative understanding of causality, including the notion of mechanisms, is not yet integrated in much of the existing integrated care studies and theoretical frameworks.^73^ For example, the theory on integration as introduced by Singer and colleagues does not explicitly acknowledge the potential role of generative mechanisms.^7^ Nevertheless, particular concepts that may be closely related to mechanisms such as norms and collective attitude are discussed in Singer's theory.^7^ Moreover, as found by Astbury and colleagues, typical RE concepts like mechanisms are still ambiguously conceptualized and used.^75^ More specifically, the key features of mechanisms—that they are usually hidden, sensitive to variations in context, and may generate outcomes—are not always acknowledged.^75^ As a result, RE studies have often conflated observable intervention modalities, modes of implementation, and activities with mechanisms.^57^, ^75^ This limits the usefulness of existing RE studies to inform future RE studies in the field of integrated care.

A second new insight for the field of integrated care, as retrieved from the presented IPT, relates to the hypothesized conditionality between context and outcomes, both within and between TARGET's program components. Singer's theory does acknowledge the relationship between context and outcomes in integrated care: the former would serve as the precursors to different types of integration and would, as such, indirectly trigger desired outcomes.^7^ TARGET's IPT adds to this existing theory by suggesting that a new context is shaped by preceding intermediate outcomes. As such, the required circumstances are shaped in which the next mechanisms can be triggered, potentially leading to the desired final outcomes. For instance, an accommodating context would be shaped by the intermediate outcome, i.e., feeling heard, of the preceding PCNA for patients. This context would serve to trigger the next mechanism: confidence to receive integrated, person‐centered care. By activating this mechanism, long‐term outcomes, i.e., improved patient experience and population health would eventually be achieved. In realist literature, this is referred to as the ripple effect.^76^ Underlying the ripple effect, there is the perception of an intervention as a “critical event in the history of a system, leading to the evolution of new structures of interaction and new shared meanings.”^76^, ^77^

Our in‐depth, theorized insight into program functioning at the developmental stages can help to shape subsequent monitoring and evaluation efforts of the TARGET program when implemented. RE takes a more neutral position toward methodology than method‐oriented approaches to evaluation.^19^, ^26^ Hence, a broad range of methods may be useful, but the chosen research methods must have the potential to substantiate the complex ICAMO in play.^26^ This implies that traditional methods, tools, and analysis techniques may need to be adjusted to ensure that the RE philosophy is adequately accommodated.^26^ An example of such an adjusted method is the realist, theory‐driven interview, introduced by Pawson.^78^ This technique requires that the interviewer first takes on a role to teach the respondent about the hypothesized program theory.^78^ Subsequently, the respondent would “teach the evaluator about those components of a programme in a particularly informed way.”^58^, ^59^, ^79^ As such, the developing program theory can be further refined. Qualitative methods are most commonly used in realist‐inspired studies in the health sector.^79^ This seems defensible, given the latent nature of mechanisms.^79^ Still, to obtain insights into all elements of an RE heuristic tool, it is suggested to not only use a mixture of different methods and methodologies, but also to collect rich data: “Substantial amounts of primary or secondary data are needed—even when the sample is small—to move from constructions to explanation of causal mechanisms.”^79^

A strength of the current study is that information was derived from multiple sources to elicit the IPT: existing theory on integration, previous studies into integrated care, and expert interviews. The different sources provided various abstract and/or tacit theoretical insights that contributed to a rich and reflective elicitation process. However, several limitations regarding the choice of data sources for IPT elicitation in this study are noteworthy as well.

Unlike other IPT elicitation studies, the current study was not informed by a realist synthesis of previous, comparable integrated care studies.^67^, ^80^ However, in different methodological steps, insights from existing integrated care literature were implicitly integrated. First, we used a widely known and applied theory on integration by Singer and colleagues, which itself is underpinned by a comprehensive synthesis of integrated care literature.^7^, ^81^ Second, existing integrated care literature, including our own studies informing TARGET, were consulted in phase 2 and helped in composing the preliminary IPT. ^30^, ^31^, ^32^, ^33^, ^34^ Third, the preliminary IPT was discussed with several experts who themselves have experience with specific programs in the field of integrated care and are informed of the functioning of comparable programs described in the literature. Given these steps, and considering the novelty of RE for the field of integrated care and prevailing misconceptions about RE concepts (in particular, mechanisms), we question to what degree a dedicated literature synthesis would have added further valid new insights to our IPT.^57^, ^75^ However, a broad literature synthesis could provide insight into promising new intervention components, resources, and related outcomes. Hence, although a realist review may not have led to significantly new insights for the IPT at this stage of the process, it may serve as a source of inspiration for the developing TARGET program and could be added in the subsequent stages of the RE process. A variety of other methods are needed in addition to this review, to ensure a reflective, robust evaluation process that addresses all different aspects of the evolving program theory. Examples are realist interviews with professionals and patients, observations of PCNAs, and analyses of PCAM results.

Another limitation pertains to the early phase, before implementation of TARGET, in which the IPT was formulated. As a result, the developing IPT could not be informed by preliminary evaluation insights—for instance, experiences of program users. Unraveling the IPT preimplementation, however, allows for subsequent evaluation efforts to explore the degree to which the hypothesized IPT is valid in practice. To this end, in‐depth theory‐driven realist interviews with program users, besides the range of other methods as described herein, will be a particularly important new source of information.

## Conclusions

Rethinking the conceptualization of causality and evaluation of complex interventions within the critical realist paradigm has paved the way for RE. Methodologically, the RE approach is useful for unraveling why and how programs work, questions that are often left unaddressed when adopting a traditional, experimental design. Furthermore, the presented IPT in this paper has shed light on new theorized insights for the field of integrated care—that is, the overarching types of mechanisms (confidence and mutual trust) as well as the conditionality between context and outcomes. Above all, unraveling a program's IPT prior to implementation can inform robust evaluation processes and maximizes the opportunity to gather transferable insights. Hence, we conclude that putting “first things first,”—that is, eliciting the IPT for a theory‐driven understanding of how and why complex programs work—is a methodological asset to the field of integrated care.

*Funding/Support*: This study was funded by the Dutch primary care group HZD and the Dutch health insurance company Zilveren Kruis (fund Stichting Achmea Gezondheidszorg; project code Z781‐2). The funding agencies had no role in study design, data collection and analysis, decision to publish, or preparation of the manuscript.

*Acknowledgments*: The authors wish to thank all experts in realist evaluation who participated in this study.

*Conflict of Interest Disclosure*: All authors completed the ICMJE disclosure form. No conflicts were reported

## Methodological Comparison of RE With Traditional Evaluation Approaches

When compared to traditional, experimental approaches to evaluation, RE has a significantly different way of preparing, conducting, and optimizing the evaluation of an innovation.^15^, ^27^ This can be traced back to a distinct philosophical standpoint and understanding of causality. A linear, successionist understanding of causality is inherent to experiments.^15^, ^27^ This implies “that causality is established when the cause X is switched on (experiment) and effect Y follows.”^27^ Within RE, the relation between what is offered and changed as part of a program and what (intendedly) comes out, is perceived as much more complex. Program effects are assumed to depend on whether the right intervention resources, within a supporting context, are able to *generate* mechanisms in the right actors. Hence, a generative notion of causality is what defines RE.^15^, ^27^ These fundamentally contrasting standpoints can be clarified and illustrated by the resulting difference in the mode of inference, as well as the use of visual representations to guide program planning and evaluation.

Induction and deduction are dominant modes of inference in scientific research.^62^ Both modes are built on the assumption that empirical observations, from which evidence can be generated, play a key role in inference making. In induction, evidence derived from “studying one or many cases”^62^ is used to come to general theories. In an opposite direction, deduction takes theory as a starting point and aims to test this theory by studying and generating evidence from specific cases.^62^ In scientific realism, these modes are criticized in the light of so‐called inference sufficiency. This means that both induction and deduction would be “insufficient for analyzing ontologically deep phenomena and risk creating scientific outputs that are ontologically flat.”^62^ Realism assumes a deeper, latent, and difficult‐to‐observe layer of mechanisms playing a key role in how behavior is changed and programs work. Thus, it is key to adopt a mode of inference suited to move beyond that empirical, objective layer of reality. Therefore, the retroduction mode of inference is advocated in realism.^22^, ^62^, ^63^, ^82^ This is referred to as “the activity of theorizing and testing for hidden causal mechanisms responsible for manifesting the empirical, observable world.”^62^ Retroduction is often alternated with and reinforced by another mode of inference called abduction. This last mode helps to creatively think about, imagine, and reconceptualize the mechanisms that were retroductively theorized.^62^

Various ways of visualizing program components, their functioning, and their intended achievements, exist. Perhaps most well known and commonly used is the logic model.^83^, ^84^, ^85^ A logic model is described as “a systematic and visual way to present and share your understanding of the relationships among the resources you have to operate your program, the activities you plan, and the changes or results you hope to achieve.”^83^ To some extent, logic models show similarities with the visual representations as composed and presented in the current RE paper. Both ways of visualizing intend to clarify and simplify how the different components of a program should be positioned in relation to each other. Doing so can help to guide and plan the evaluation process. However, there are also important differences that reflect a different way of looking at an innovation, what defines its success, and how an evaluation process should be designed.

First, logic models present all kinds of program components (e.g., resources, activities, outputs) that are tangible and can be measured and monitored empirically and objectively. For instance, in a recently developed logic model for an integrated care program, different partnerships are identified as important inputs, without explicating the mechanisms and contextual influences determining the success of these inputs.^85^ RE representations present a deeper, latent layer of generative mechanisms, which are positioned at the heart of program functioning, hence the visual representation.

Second, while evaluation efforts preceding and following development of a logic model may take into account the embeddedness of an intervention into an existing and dynamic context, contextual influences are not always explicitly visually included. In RE, however, context is perceived as an overarching layer that must never be overlooked, as it determines whether mechanisms fire or not.

And third, logic models are presented in a linear way. Inputs, activities, outputs, outcomes, and impact are positioned in a straight line, and models should be “read from left to right.”^83^ The RE visualizations highlight the complex, nonlinear functioning of a program and the assumption that the outcomes of one program mechanism can lead to changed contexts in which new mechanisms may be triggered, referred to as the ripple effect.^76^
